# Distributed circuits underlying anxiety

**DOI:** 10.3389/fnbeh.2014.00112

**Published:** 2014-04-01

**Authors:** Avishek Adhikari

**Affiliations:** Deisseroth Laboratory, CNC Program, Bioengineering Department, Stanford UniversityPalo Alto, CA, USA

**Keywords:** anxiety, amygdala, BNST, ventral hippocampus, medial prefrontal cortex

## Abstract

Anxiety is of paramount importance for animals, as it allows assessment of the environment while minimizing exposure to potential threats. Furthermore, anxiety disorders are highly prevalent. Consequently, the neural circuitry underlying anxiety has been a topic of great interest. In this mini review, we will discuss current views on anxiety circuits. We will focus on rodent anxiety paradigms, but we will also consider results from human neuroimaging and clinical studies. We briefly review studies demonstrating the central role that the amygdala and the bed nucleus of the stria terminals (BNST) play in modulating anxiety and present evidence showing how the bed nucleus uses different output pathways to influence specific features of anxiolysis. Lastly, we propose that several brain regions, such as the medial prefrontal cortex (mPFC) and the ventral hippocampus (vHPC), act in a coordinated fashion with the amygdala and BNST, forming a distributed network of interconnected structures that control anxiety both in rodents and humans.

## Introduction

In the clinical literature anxiety is defined as a long-term trait characterized by non-adaptive hypervigilance and overestimation of the potential for threat in uncertain situations (Sylvers et al., 2011). On the other hand, in the animal literature anxiety is often defined as a temporary behavioral state induced by diffuse threatening stimuli (Sylvers et al., 2011), such as open spaces (Pellow et al., 1985) and bright lights (Crawley, 1985). Despite these differences, similar brain structures underlie both human (Yassa et al., 2012; Boehme et al., 2013) and rodent (Moreira et al., 2007; Duvarci et al., 2009) measures of anxiety, and drugs that are anxiolytic in humans decrease avoidance towards open spaces and bright lights in rodents (File and Pellow, 1985; Walker and Davis, 1997a; Schmitt and Hiemke, 1998). These similarities strongly suggest that the study of rodent anxiety paradigms can provide insights into human anxiety disorders.

Considering anxiety disorders generate large financial and emotional burdens (Lecrubier, 2007), it is unsurprising that the circuits underlying anxiety have received tremendous attention. Here, we will cover a few topics relating to anxiety. We will first introduce common rodent anxiety paradigms and discuss the role of the amygdala and the bed nucleus of the stria terminalis (BNST) in anxiety. Then, we will discuss evidence showing that the BNST and the amygdala are part of a larger network that modulates anxiety, involving the ventral hippocampus (vHPC), the medial prefrontal cortex (mPFC) and other regions.

## Animal models of anxiety

In humans anxiety is assessed by self-report. While this method cannot be used in rodents, one can measure their innate avoidance of bright lights and open spaces. Rodents avoid open areas and brightness presumably because they are more vulnerable to predators. Avoidance towards openness is commonly studied in the elevated plus maze, a paradigm consisting of a plus-shaped maze on an elevated platform. It contains two open arms without walls and two closed arms which are enclosed by high walls. Rodents innately avoid the open arms (Pellow et al., 1985). Another similar paradigm is the open field, which consists of an enclosure with high walls. As expected, rodents spend more time exploring the walled periphery of the open field and avoid its exposed center. This avoidance behavior has pharmacological validity, as drugs that decrease anxiety in humans, such as benzodiazepines, decrease the aversion rodents have towards open spaces (File and Pellow, 1985; Schmitt and Hiemke, 1998).

Rodents also show innate aversion to bright lights. This behavior has been studied using the startle paradigm, in which rodents display a startle reflex when presented with an unpredictable burst of noise. Interestingly, the amplitude of the startle is enhanced after rats are presented with an innately aversive stimulus, such as a cat (Blundell et al., 2005) or bright illumination (Walker and Davis, 1997a). This paradigm has been validated pharmacologically, as the amplitude of the light-potentiated startle is decreased by the anxiolytic drug buspirone (Walker and Davis, 1997a). While there are other paradigms that measure different types of anxiety, such as social anxiety (Pobbe et al., 2011), here, we will focus on paradigms measuring aversion towards open spaces and bright lights as these are among the most common anxiety paradigms.

## The amygdala in anxiety

A wealth of data implicates the amygdala in anxiety both in humans and rodents. For example, higher amygdala volume is correlated with more anxiety in humans (Qin et al., 2013; Machado-de-Sousa et al., 2014), and patients with social anxiety disorder show increased amygdala activation during anticipatory anxiety relative to healthy subjects (Boehme et al., 2013). Moreover, immediate early gene assays in rodents show amygdala activation following exposure to anxiogenic contexts (Silveira et al., 1993; Butler et al., 2012), and pharmacological inactivation of the amygdala is anxiolytic in the elevated plus maze (Moreira et al., 2007). Thus, both human and rodent studies show that the amygdala is a crucial node in the anxiety circuitry.

The most studied amygdala sub-regions are the basolateral amygdala (BLA) and the central nucleus of the amygdala. Results from the fear conditioning literature show the BLA integrates highly processed information about the environment and encodes behaviorally relevant cues (LeDoux, 2000). Indeed, the BLA has cells that respond to cues that predict threats (Amano et al., 2011) and cells that respond to cues that no longer predict danger (Senn et al., 2014). The BLA robustly projects to the central nucleus of the amygdala (Pitkanen, 2000; Tye et al., 2011) and its main excitatory input (LeDoux, 2000). The central nucleus of the amygdala is comprised of central lateral and central medial portions. The central lateral amygdala inhibits the central medial nucleus (Jolkkonen and Pitkänen, 1998; Tye et al., 2011), which is the main output of the amygdala (LeDoux, 2000). A crucial role for the central nucleus of the amygdala in mediating behaviors induced by threatening stimuli was demonstrated by a study showing decreased freezing to a tone that predicts a shock following central medial nucleus inactivation (Ciocchi et al., 2010). This effect is presumably mediated by projections of the central medial nucleus to hypothalamic and brain stem targets (Price and Amaral, 1981) which modulate various features of the anxious state.

Local pharmacological inactivation and lesion studies indicate that the central nucleus of the amygdala, but not the BLA, is required for avoidance of open spaces (Moller et al., 1997; Moreira et al., 2007; Carvalho et al., 2012). However, these results may reflect compensatory changes after drug infusions or lesions. Fortunately, optogenetics allows researchers to overcome these limitations as optogenetic manipulations are quick and reversible, and are thus less likely to be confounded by slow compensatory changes. It was shown that optogenetic activation of the entire BLA augments anxiety, while selective activation of the projection from the BLA to the central lateral nucleus decreases anxiety (Tye et al., 2011). These results make anatomical sense, as the central lateral nucleus inhibits the central medial nucleus (Tye et al., 2011), which is the main output structure of the amygdala (Price and Amaral, 1981). These data indicate that classifying an entire amygdala region as anxiogenic or anxiolytic is an oversimplification, as different cells in the same region can have different functions depending on their post-synaptic targets (Tye et al., 2011).

It is noteworthy that the amygdala has other regions besides the BLA and the central nucleus of the amygdala, which also mediate defensive behaviors. For example, the medial amygdala is required to react to olfactory cues from a predator (Li et al., 2004), whereas the basomedial amygdala mediates avoidance of potentially threatening auditory and visual cues (Gross and Canteras, 2012).

In summary, prior reports demonstrate the amygdala is crucial for generating anxiety. However, some sub-regions have been more extensively studied than others. Furthermore, the study of functional differentiation among subpopulations of cells in the same amygdala region has only just begun.

## Beyond the amygdala: the extended amygdala

The prominent role of the amygdala in mediating anxiety motivated researchers to identify other amygdala-associated structures that also influence anxiety. Anatomical studies suggest the BNST modulates anxiety, as it receives prominent projections from the amygdala (De Olmos, 1972; Dong et al., 2001a) and projects to many hypothalamic and brainstem structures (De Olmos and Ingram, 1972; Holstege et al., 1985; Dong et al., 2001b; Dong and Swanson, 2004) that receive central amygdala (CeA) nucleus terminals (De Olmos, 1972; LeDoux, 2000). Furthermore, the central nucleus of the amygdala and the BNST are also similar in their neuropeptide expression profile (Roberts et al., 1982; Woodhams et al., 1983) and morphology (McDonald, 1983). Noting these similarities, Alheid et al proposed that the BNST is part of the extended amygdala, a group of anatomically and functionally related structures which include the BNST and the central nucleus of the amygdala (Alheid and Heimer, 1988; Alheid et al., 1998).

Like the amygdala, a large body of evidence implicates the BNST in anxiety. For example, compared to control subjects, patients with generalized anxiety disorder show hyperactivation of the BNST when participating in a gambling task with high uncertainty (Yassa et al., 2012). Moreover, the BNST is recruited during hypervigilance in individuals with higher trait-anxiety (Somerville et al., 2010). Thus, human imaging studies suggest that BNST activity is correlated with increased anxiety. The rodent literature, however, presents conflicting results. For example, Treit et al. (1998) found no effect of BNST lesions in open-arm avoidance in the elevated plus maze, whereas Duvarci et al. (2009) reported anxiogenic effects of BNST lesions on the plus maze. Furthermore, van Dijk et al. found that electrical stimulation of the BNST did not alter behavior in the plus-maze (van Dijk et al., 2013), and Walker reported that infusions of glutamate antagonists in the BNST are anxiolytic in the light potentiated startle paradigm (Walker and Davis, 1997b). These data show the BNST modulates anxiety in rodents, but they do not make it clear if this structure increases or decreases anxiety.

The anatomy of the BNST provides a potential explanation for these discrepancies. Anatomists have recognized that the BNST is composed of several sub-nuclei differing in anatomical (Dong et al., 2001b; Dong and Swanson, 2004) and neurochemical features (Walter et al., 1991), which likely reflect functional differentiation among BNST nuclei. If different BNST subregions regulate anxiety in opposite directions, lesion and pharmacology studies affecting distinct subregions of the BNST could provide conflicting results. Supporting this view, Kim et al. showed that optogenetic inactivation of the oval nucleus of the BNST is anxiolytic, while decreases in activity in the anterodorsal BNST (adBNST) are anxiogenic (Kim et al., 2013). These results demonstrate that different subregions of the BNST have different functions, and suggest an explanation for the discrepancies found between previous studies (Treit et al., 1998; Duvarci et al., 2009).

BNST cells may also differ in their neurochemical profiles (Walter et al., 1991), suggesting functional differentiation among cells located in the same BNST nucleus. Indeed, Jennings et al. (2013b) showed that the firing rate of BNST glutamatergic neurons increased when mice were presented with aversive stimuli, whereas BNST GABAergic neurons decreased firing in the same condition. Furthermore, optogenetic activation of the projection from glutamatergic BNST cells to the ventral tegmental area (VTA) was anxiogenic and aversive, while activation of the projection of GABAergic BNST cells to the VTA was anxiolytic and rewarding (Jennings et al., 2013b). A role for the BNST in reward is also suggested by its involvement in stress-induced reinstatement of cocaine seeking (Erb and Stewart, 1999). This finding may partially explain the correlation between trait anxiety and severity of drug addiction (O’Leary et al., 2000). Lastly, it has also been shown that activation of axon terminals of GABAergic BNST neurons in the lateral hypothalamus robustly increases eating in mice (Jennings et al., 2013a). This effect may play a role in the weight and feeding abnormalities seen in patients with anxiety disorders (Deboer and Smits, 2013). These results show that the neurochemical identity of BNST neurons is reflected in important functional differences. It is noteworthy that various neuropeptides, such as enkephalin (Arluison et al., 1990), neuropeptide Y and substance P (Walter et al., 1991) are richly expressed in this structure. Future studies are needed to identify the role of these neurotransmitters in anxiety.

Taken together, these data show that subregion specificity, neurochemical composition and anatomical connectivity are all features to be considered when studying the BNST. Indeed, optogenetic manipulation of specific anatomical projections, such as activation of BNST projections to the VTA (Jennings et al., 2013b) and of BLA terminals in the BNST (Kim et al., 2013) modulated anxiety, suggesting that a network of several brain regions acts together to dynamically fine-tune the expression of different features of anxiety according to ever-changing environmental demands.

## A distributed network underlying anxiety

The view that a distributed network of interconnected brain regions modulates anxiety is not new and has been suggested previously (Papez, 1995). Here, we will briefly review recent evidence supporting this view, focusing on rodent *in vivo* electrophysiology papers, projection-targeting optogenetic studies, and neuroimaging reports.

Rodent *in vivo* recordings suggest the interplay between the mPFC, the BLA, and the vHPC affects anxiety. The first *in vivo* recording supporting this idea came from a fear conditioning study showing that BLA-hippocampus synchrony increases while animals freeze to a shock-predicting auditory tone (Seidenbecher et al., 2003). More recently, it has been shown that mPFC neurons encode arm type in the plus maze, by being preferentially active in either the closed or open arms, and the neurons that encode arm type most strongly also are more synchronized to vHPC activity (Adhikari et al., 2011). This result suggests the vHPC-mPFC pathway encodes aspects of the context relevant to anxiety. Furthermore, neural synchrony between the vHPC and the mPFC in the θ-range (4–12 Hz) increases while mice explore the elevated plus maze or the open field compared to a familiar environment (Adhikari et al., 2010). Additionally, in the open field the amount of increase in mPFC-BLA θ-range synchrony correlates with higher avoidance of the center of the open field (Likhtik et al., 2014). Lastly, mPFC-BLA and vHPC-mPFC synchrony increase before the animal transitions from dangerous to safe zones in anxiety paradigms (Adhikari et al., 2010; Likhtik et al., 2014), suggesting synchrony between these regions could influence exploration in an anxiogenic environment. In summary, rodent electrophysiology studies indicate a network of structures modulates anxiety.

However, these data are correlative and cannot demonstrate whether interactions among these brain regions affect anxiety. Perturbative methods are needed to investigate this question. To this end, it has been shown that local injections of gap junction blockers in the vHPC reduced both vHPC-mPFC synchrony and anxiety in the elevated plus maze and open field (Schoenfeld et al., 2013). The role of the BLA-vHPC pathway has also been studied through perturbative methods. It has been reported that optogenetic activation of the BLA-vHPC projection increases anxiety, whereas inhibition of this projection was anxiolytic in the elevate plus maze and open field (Felix-Ortiz et al., 2013). Just as the BLA, the BNST also has a prominent role in the anxiety network. Optogenetic activation of the adBNST-lateral hypothalamus projection was anxiolytic in the plus maze and open field, whereas activation of the adBNST-parabrachial nucleus (PB) projection selectively decreases respiration rate (a physiological marker of anxiety), but not behavior. Lastly, optogenetic activation of the adBNST-VTA pathway selectively induces conditioned place preference, as mice spend more time in a compartment in which the adBNT-VTA projection was activated (Kim et al., 2013). These data show different outputs from the BNST control distinct features of anxiolysis. Thus, both rodent *in vivo* physiology and optogenetic studies suggest several interconnected regions influence anxiety.

**Figure 1:**
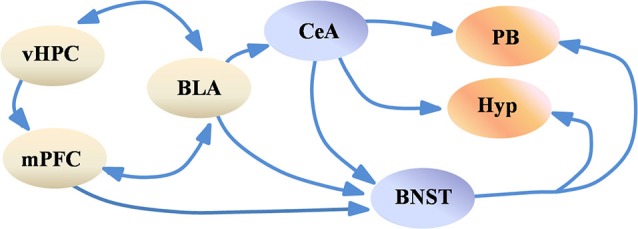
**Simplified scheme of the anxiety network**. Highly processed sensory and contextual information from the vHPC and the mPFC is integrated by the BLA, which in turn may activate the CeA and the BNST. The CeA and the BNST project to the hypothalamus and to brain stem nuclei such as the PB, which modulate various features of anxiety, such as avoidance of open spaces and changes in respiration rate. For simplification purposes subregions of the BNST, CeA, Hypothalamus and mPFC are not shown. Abbreviations: BLA: basolateral amygdala, BNST: bed nucleus of the stria terminalis, CeA: central amygdala, Hyp: hypothalamus, mPFC: medial prefrontal cortex, PB: parabrachial nucleus, vHPC: ventral hippocampus.

Evidence from human neuroimaging studies also support this view. For example, co-activation of the amygdala and the insular cortex has been reported during negative affective states (Kober et al., 2008). This work also identified the periaqueductal gray, the hypothalamus and the amygdala as part of a limbic network in which members are co-activated, and which may be linked to the generation of negative valence. The authors also identify the hippocampus and the mPFC as part of a separate co-activated network, which can modulate and be modulated by the limbic network. Lastly, similar to results in rodents, activity in the anterior hippocampus (the human analog of the rodent vHPC) may have a role in anxiety, as its activity is correlated with trait anxiety (Satpute et al., 2012). These data suggest that the brain structures regulating anxiety are well-conserved between rodents and humans and indicate that a distributed network modulates anxiety (Figure 1). Although the precise role of each structure in the network is not known, one possibility is that contextual and sensory input from the mPFC and the vHPC is integrated by the BLA, which then drives the central nucleus of the amygdala and the BNST. These two structures, in turn, may activate downstream regions which control anxiety-related symptoms.

Importantly, prior studies have implicated other regions in anxiety, such as the medial septum. It has been shown that septal inactivations or lesions decrease anxiety in the plus maze (Menard and Treit, 1996; Degroot and Treit, 2004). It is possible that the effect of medial septal lesions on anxiety is mediated by disruption of θ-oscillations, as the medial septum controls hippocampal θ-rhythms (Smythe et al., 1992). θ-activity is strongly implicated in anxiety, as θ-range synchrony between the vHPC, mPFC and BLA is seen during high anxiety in rodents (Adhikari et al., 2010; Likhtik et al., 2014), and all clinically effective anxiolytics inhibit θ-activity (McNaughton and Gray, 2000). Other regions implicated in anxiety include the insula (Kober et al., 2008), the nucleus basalis magnocellularis (Privou et al., 1998) and the ventral striatum (Kabli et al., 2013), among others. Future studies will identify all the components of this network and dissect the function of each projection. Lastly, one must consider that even though anxiety consists of multiple features (behavioral, hormonal, etc.), other equally complex constructs are influenced by anxiety through unknown mechanisms. These include aggression (Tang et al., 2013), risk-taking (de Visser et al., 2010) and feeding (Deboer and Smits, 2013). Investigating these outstanding questions may provide insights leading to novel therapies for anxiety disorders.

## Author contributions

Avishek Adhikari wrote the manuscript.
